# Recurrent Ewing’s Sarcoma of the Chest Wall in an Adolescent Male Patient: A Complex Multimodal Management and Progressive Disease Course

**DOI:** 10.7759/cureus.98192

**Published:** 2025-11-30

**Authors:** Asmaa M AlRefaie, Salma Almarwani, Renad Alshaikh, Muḥammad Alfattani

**Affiliations:** 1 Department of Family Medicine, National Guard Hospital, Jeddah, SAU; 2 College of Medicine, King Saud Bin Abdulaziz University for Health Sciences, Jeddah, SAU; 3 Department of Pediatrics, Maternity and Children's Hospital, Makkah, SAU

**Keywords:** adolescent oncology, chemotherapy resistance, recurrence, supportive and palliative care, thoracic ewing's sarcoma

## Abstract

Ewing’s sarcoma is a rare, aggressive malignant tumor of bone and soft tissue that predominantly affects adolescents and young adults. Chest wall involvement, though uncommon, presents unique surgical and oncologic challenges. We report a 17-year-old male patient who initially presented with thoracic pain and swelling. Imaging revealed a posterior chest wall mass consistent with Ewing’s sarcoma. He underwent surgical resection with rib removal, followed by multiple lines of chemotherapy (vincristine, adriamycin (doxorubicin), and cyclophosphamide/ifosfamide and etoposide (VAC/IE) ×15 cycles; cyclophosphamide/topotecan ×4 cycles; etoposide, vincristine, adriamycin (doxorubicin), ifosfamide, and actinomycin D (EVAIA) ×2 cycles; and gemcitabine/docetaxel ×3 cycles). Despite aggressive multimodal therapy, the disease demonstrated rapid progression with extensive local invasion, spinal canal involvement, and pleural metastases. The patient ultimately developed systemic progression with recurrent infections and succumbed to his disease in August 2024. This case highlights the aggressive nature and chemoresistance of recurrent Ewing’s sarcoma, emphasizing the importance of multidisciplinary care and early palliative involvement in advanced stages.

## Introduction

Ewing’s sarcoma is a highly malignant small round-cell tumor characterized by aggressive local invasion and early metastatic potential. It primarily affects long bones and the pelvis but may also arise in the chest wall, where the entity is specifically termed Askin’s tumor [1]. The disease predominantly affects adolescents and young adults, with most cases occurring before the age of 20, and it accounts for approximately 4% of pediatric malignancies. Despite its rarity, Ewing’s sarcoma carries substantial clinical impact due to its rapid progression, high recurrence rate, and the intensive multimodal therapy required for management [2]. Standard treatment consists of neoadjuvant chemotherapy, wide surgical resection, and adjuvant radiotherapy, an approach that has significantly improved outcomes over recent decades [1,2]. However, recurrence remains a major therapeutic challenge. Patients with chest wall primaries face particularly poor prognoses because complete margin-negative resection is often difficult due to the proximity of tumors to vital mediastinal structures [1,3]. In addition, data from the Euro-EWING trials and subsequent multicenter analyses have shown that recurrent or refractory disease is associated with markedly inferior survival, reflecting both biological aggressiveness and therapeutic resistance [4,5]. Recent studies demonstrate that relapsed Ewing’s sarcoma often exhibits chemoresistance and follows heterogeneous relapse patterns, especially when the initial disease presents with extensive pulmonary or pleural involvement [6,7]. Prognostic factors, such as initial tumor volume, metastatic burden, time to relapse, and molecular features including EWS-FLI1 fusion subtype, continue to inform risk stratification but remain insufficient to reliably guide salvage therapy selection [7,8]. This report describes a rapidly progressive and chemoresistant chest wall Ewing’s sarcoma in an adolescent male who underwent multiple evidence-based therapeutic strategies. Despite timely diagnosis, aggressive resection, and adherence to standard systemic and radiotherapeutic protocols, the tumor demonstrated relentless local and metastatic progression. The case highlights not only the limitations of currently available treatments for recurrent Ewing’s sarcoma but also the complex decision-making required when curative options are exhausted. It further underscores the importance of early palliative care integration to support patients and families in advanced stages of pediatric oncology.

## Case presentation

Initial presentation and diagnostic workup

**Figure 1:**
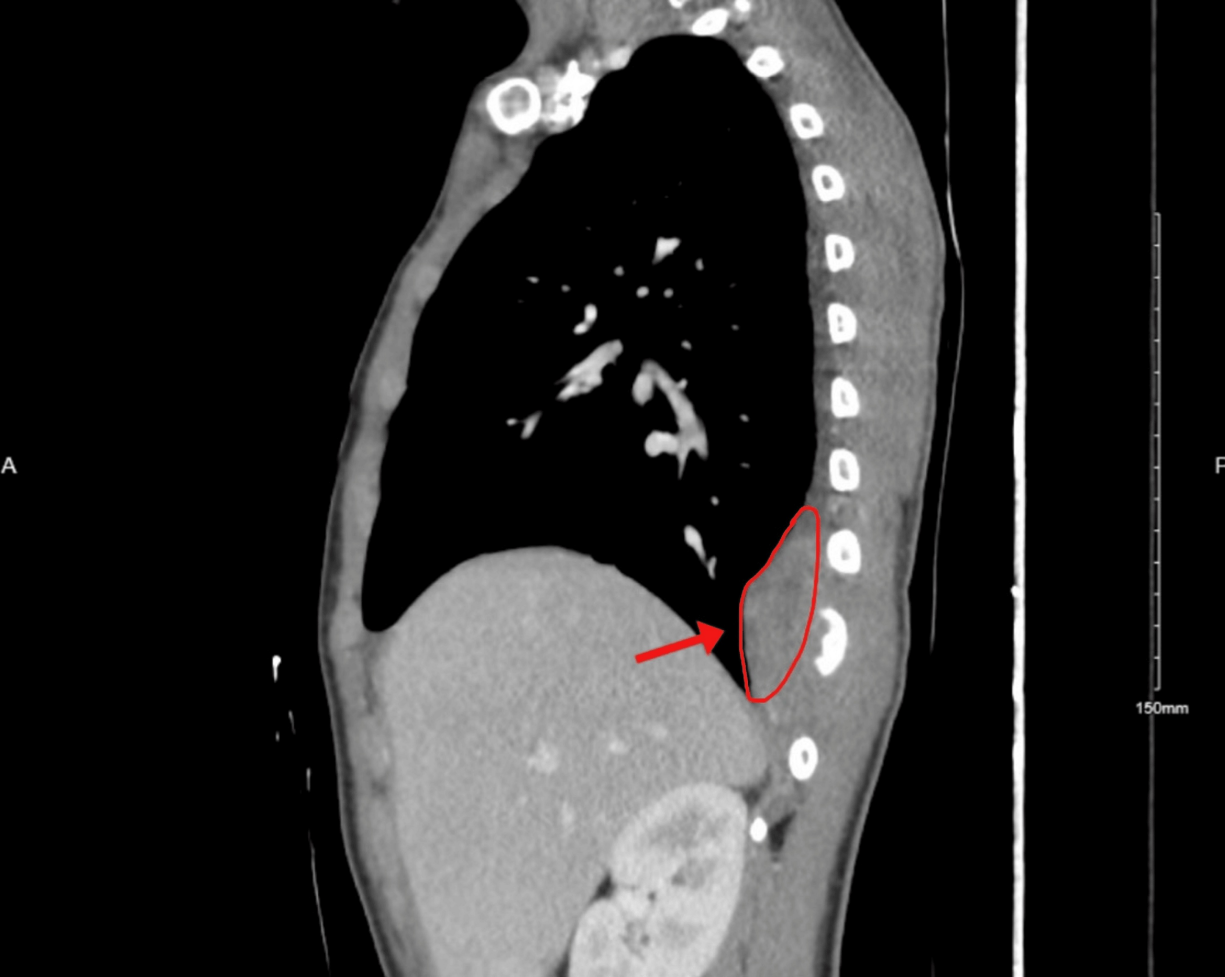
Chest CT demonstrating posterior chest wall lesion

**Figure 2:**
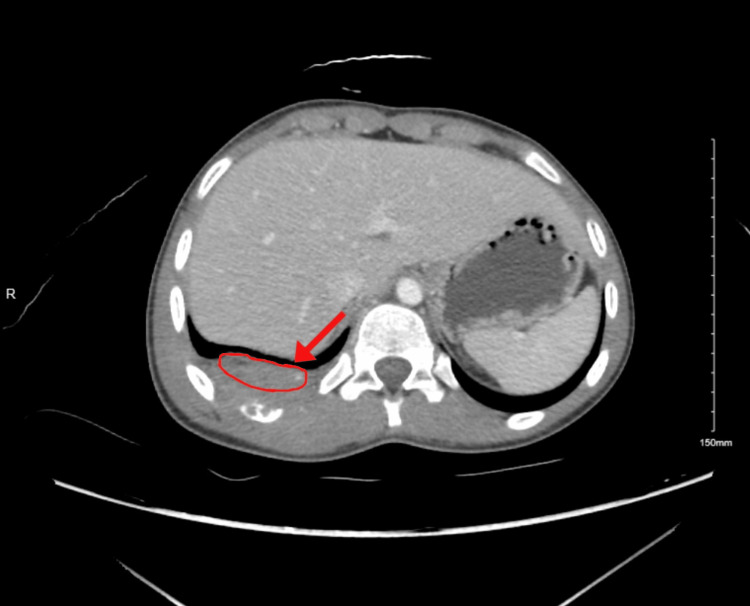
Abdominal CT of posterior chest wall mass consistent with Ewing’s sarcoma

In November 2022, a 15-year-old boy presented to the Family Medicine Clinic with a seven-month history of right mid-thoracic pain, worsened by deep inspiration. His past history was significant for two years of growth hormone therapy. Examination revealed localized tenderness over the 7th-8th ribs. He was referred to Thoracic Surgery, where evaluation identified a left upper back swelling without overlying skin changes. Initial staging imaging included a contrast-enhanced chest CT, which demonstrated a posterior chest wall mass arising from the ribs and extending into the paraspinal soft tissues (Figures 1-2). A CT of the abdomen and pelvis, and a CT of the spine were also performed as part of metastatic staging and showed no evidence of distant disease at that time. A core needle biopsy of the chest wall mass confirmed Ewing’s sarcoma, demonstrating sheets of small round blue cells with strong CD99 membranous positivity. Molecular testing identified the EWSR1-FLI1 translocation, consistent with classic Ewing sarcoma biology. Regarding the interval from symptom onset to diagnosis: although the patient reported symptoms as early as April 2022, the first medical evaluation occurred in November 2022, and a definitive diagnosis was established shortly thereafter, following imaging and biopsy. Treatment initiation in June 2023 represented a multistep process that included referral to subspecialty care, tissue diagnosis, tumor board review, and arranging for surgical resection. However, this time frame is still longer than typically expected in pediatric oncology, and the delay may have contributed to the extent of disease at presentation. 

Diagnosis and initial management

In June 2023, the patient underwent posterior chest wall resection with en bloc removal of the 10th rib. Postoperatively, he received 15 cycles of vincristine, adriamycin (doxorubicin), cyclophosphamide/ifosfamide, and etoposide (VAC/IE) chemotherapy. Persistent and progressive disease on interval imaging prompted a switch to cyclophosphamide/topotecan, which he received for four cycles.

Recurrence and subsequent treatments

By January 2024, surveillance CT demonstrated a significant recurrence of the posterior chest wall tumor with increasing soft tissue extension. He was started on etoposide, vincristine, adriamycin (doxorubicin), ifosfamide, and actinomycin D (EVAIA) chemotherapy, but follow-up scans showed further growth. The case was reviewed in a multidisciplinary sarcoma tumor board, which recommended salvage systemic therapy. He was transitioned to gemcitabine + docetaxel in May-June 2024, with initial clinical stability. In July 2024, he presented with yellowish drainage from the tumor site, tachycardia, and cellulitis. Ultrasound demonstrated superficial and deep fluid collections, and he was treated with piperacillin-tazobactam.

Advanced disease, palliative care, and outcome

**Figure 3:**
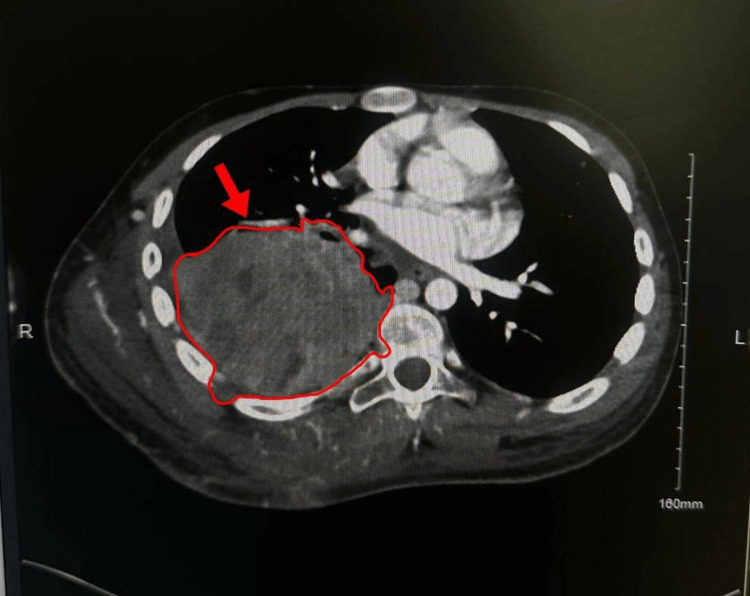
An axial contrast-enhanced CT of the thorax showing the large heterogenous right posterior-inferior chest-wall mass compressing the right atrium and extending into the spinal canal at T12/L1

**Figure 4:**
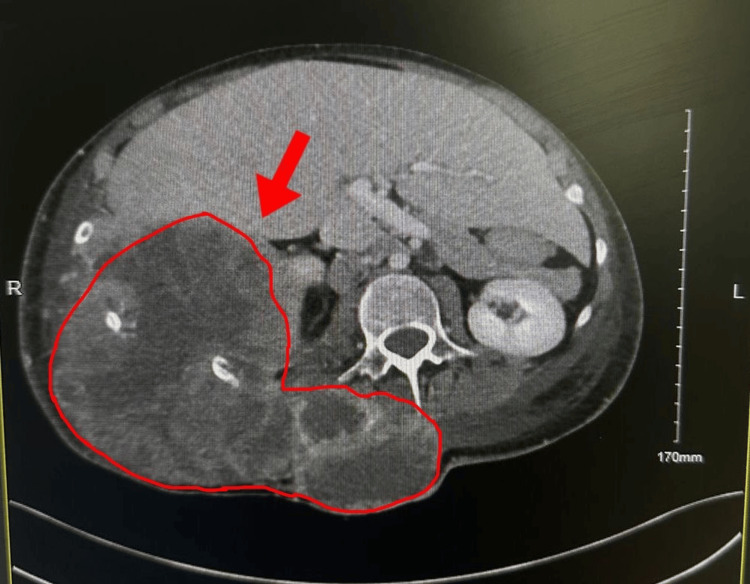
An abdominal CT image with inferior tumor extension abutting the liver and right kidney

**Figure 5:**
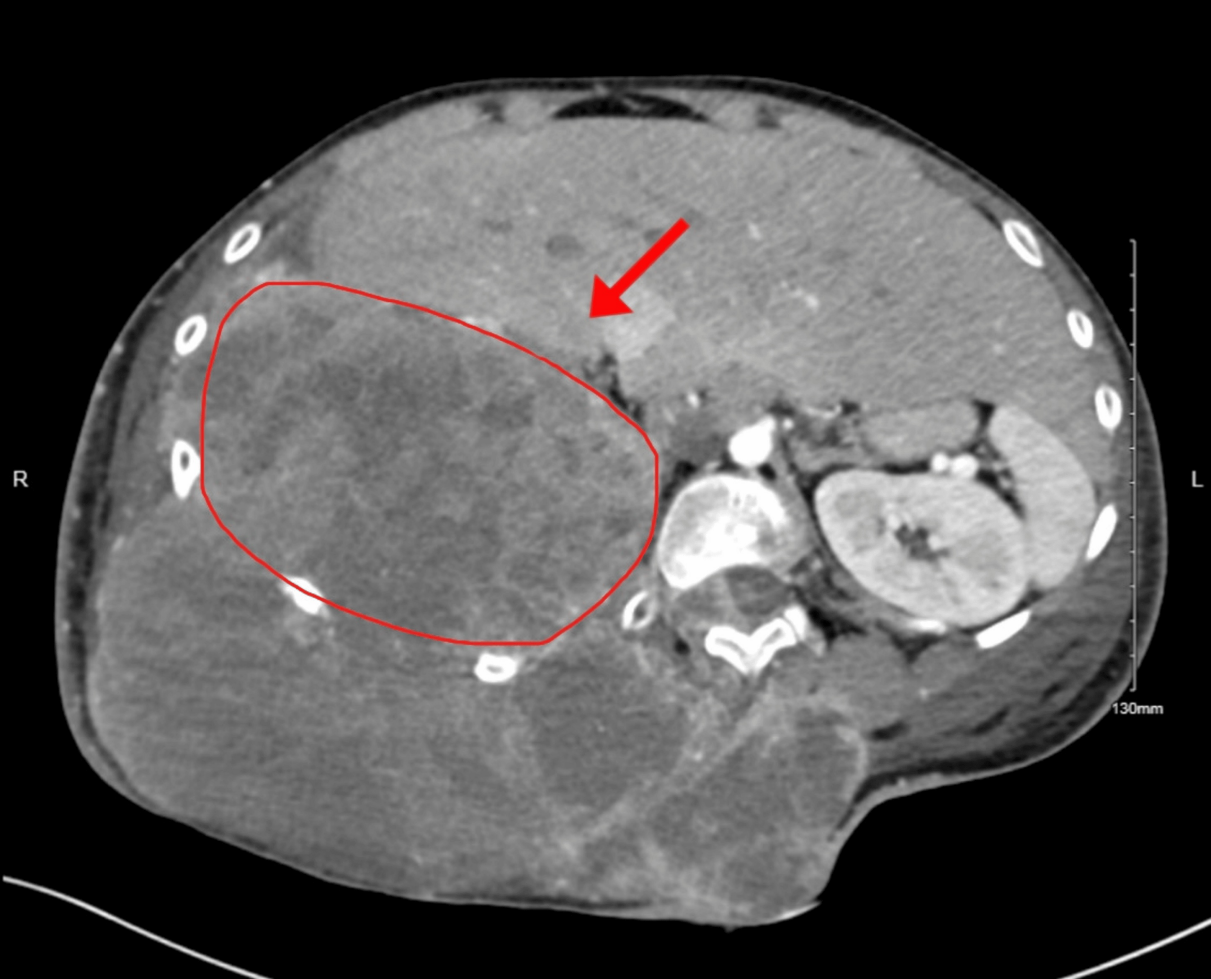
Tthe abdominal CT image demonstrating further inferior extension of the mass into the upper abdomen

Repeat CT in August 2024 showed massive tumor progression (23 × 20 cm), with invasion of ribs 9-11, epidural extension at T12-L1, and compression of the right atrium and inferior vena cava (IVC). New axillary, hilar, and subcarinal lymphadenopathy and small bilateral pleural effusions were present. He received palliative radiotherapy (30 Gy in 10 fractions) and broad-spectrum antibiotics (meropenem + vancomycin) for recurrent infections. Neurosurgery found no operable targets. Palliative Care and Psychiatry managed his pain, mood, and symptoms with Tylenol #3 and oral morphine. Despite aggressive supportive care, the patient developed progressive cachexia, recurrent infections, and respiratory compromise. He passed away in August 2024 due to progressive metastatic Ewing’s sarcoma (Figures 3-5).

## Discussion

Ewing’s sarcoma of the chest wall presents unique management challenges due to both its aggressive biology and the anatomical constraints imposed by proximity to vital mediastinal structures [1,2]. Chest wall tumors, particularly those arising from ribs or paraspinal regions, often demonstrate extensive local invasion at the time of diagnosis, rendering complete margin-negative resection difficult and contributing to higher rates of recurrence compared with extremity primaries [1,4]. Standard treatment involves multimodal therapy, most commonly systemic chemotherapy, wide local excision when feasible, and adjuvant radiotherapy, which has historically improved survival outcomes in localized disease [2,3,5]. However, recurrence remains a major clinical obstacle. Relapsed Ewing’s sarcoma continues to be associated with poor prognosis even in the modern treatment era. Prior studies have demonstrated median post-relapse survival of less than one year, particularly in patients with early relapse, extrapulmonary metastases, or poor response to first-line therapy [5,6,8]. In the Euro-EWING 99 analysis, patients with disseminated or multifocal relapse had dismal long-term outcomes despite aggressive systemic regimens [4]. More recent data have reinforced these findings and highlight the limited durability of salvage therapies in recurrent disease [6,8]. The rapid progression seen in this patient, despite three lines of evidence-based chemotherapy (VAC/IE, cyclophosphamide/topotecan, and gemcitabine/docetaxel), reflects the treatment-resistant nature of recurrent chest wall Ewing’s sarcoma. Chemoresistance has been linked to persistent activity of the EWS-FLI1 fusion oncogene, alterations in DNA damage response pathways, and increased drug efflux pump expression, all of which contribute to decreased sensitivity to cytotoxic regimens [2,5,7]. Even emerging multi-agent salvage approaches rarely achieve durable control once metastatic spread or extensive local recurrence is established. Surgical intervention plays a central role in the curative management of localized Ewing’s sarcoma; however, its utility diminishes substantially in the setting of bulky recurrent or metastatic disease [1,2]. In this case, tumor invasion into the ribs, epidural space, and major mediastinal structures rendered repeat surgical resection nonbeneficial. Similarly, while radiotherapy is an important modality for local control and palliation, evidence suggests limited impact on long-term survival once widespread progression has occurred [3,7]. Given these limitations, early integration of palliative care is increasingly recognized as essential for patients with relapsed or refractory Ewing’s sarcoma [2,8]. Palliative radiotherapy, multimodal analgesia, psychosocial support, and infection management can significantly improve quality of life even when disease-directed treatment is no longer effective. In this patient, prompt initiation of palliative measures helped address pain, infection, and emotional distress as the disease entered a terminal phase marked by cachexia, respiratory compromise, and rapidly advancing metastases. Ultimately, this case highlights the profound challenges of managing recurrent chest wall Ewing’s sarcoma and underscores the urgent need for more effective systemic therapies, earlier identification of chemoresistance, and continued development of targeted approaches guided by fusion-driven molecular biology. It also illustrates the critical role of early, proactive palliative care in maintaining dignity and comfort for pediatric and adolescent patients facing terminal disease.

## Conclusions

This case illustrates the fulminant, rapidly progressive, and refractory nature of recurrent Ewing’s sarcoma in an adolescent patient, demonstrating how quickly the disease can advance despite adherence to aggressive, multimodal, evidence-based management. Although coordinated care involving oncology, surgery, radiotherapy, infectious disease, and palliative medicine was delivered throughout the patient’s illness, outcomes remain dismal once chemoresistance emerges, particularly in chest wall primaries where anatomical constraints limit local control. The clinical course also underscores the prognostic significance of molecular drivers such as the EWS-FLI1 fusion, which contributes to treatment resistance and unfavorable relapse patterns. As seen in this case, even multiple lines of systemic therapy, timely surgical intervention, and salvage regimens were unable to alter the disease trajectory once widespread recurrence occurred. The patient’s decline and ultimate death emphasize the urgent need for improved therapeutic strategies for relapsed Ewing’s sarcoma, including early incorporation of molecular profiling, development of targeted and fusion-directed agents, and exploration of immunotherapeutic approaches that may overcome chemoresistance. Equally important is the continued prioritization of early palliative involvement to optimize symptom control, psychosocial support, and quality of life for patients and families facing incurable disease.

## References

[1] Van Mater D, Wagner L (2019). Management of recurrent Ewing sarcoma: challenges and approaches. Onco Targets Ther.

[2] Bernstein M, Kovar H, Paulussen M, Randall RL, Schuck A, Teot LA, Juergens H (2006). Ewing's sarcoma family of tumors: current management. Oncologist.

[3] Koontz BF, Clough RW, Halperin EC (2006). Palliative radiation therapy for metastatic Ewing sarcoma. Cancer.

[4] Ladenstein R, Pötschger U, Le Deley MC (2010). Primary disseminated multifocal Ewing sarcoma: results of the Euro-EWING 99 trial. J Clin Oncol.

[5] Gaspar N, Hawkins DS, Dirksen U (2015). Ewing sarcoma: current management and future approaches through collaboration. J Clin Oncol.

[6] Xu J, Zhi X, Xie L, Sun X, Liu X, Liu K, Guo W (2022). Long-term outcome and relapse patterns in Ewing sarcoma patients with extensive lung/pleural metastases after a complete response to systemic therapy. BMC Cancer.

[7] Zöllner SK, Amatruda JF, Bauer S (2021). Ewing sarcoma-diagnosis, treatment, clinical challenges and future perspectives. J Clin Med.

[8] Arafah O, Hegazy RR, Ayadi ME, Nasr AM, Fawzy M (2024). Prognostic factors and outcome of relapsed/progressive pediatric Ewing sarcoma: single-center 10-year experience. J Egypt Natl Canc Inst.

